# Report of the Committee of the Royal Medical and Chirurgical Society, on the Subject of Suspended Animation

**Published:** 1862-09

**Authors:** 


### REPORT OF THE COMMITTEE OF THE ROYAL MEDICAL AND CHIRURGICAL SOCIETY, ON THE SUBJECT OF SUSPENDED ANIMATION.

The inquiry was conducted—

By means of experiments upon living animals;

By means of experiments upon the dead human body.

In investigating anew the subject of apnœa by means of experiments on the lower animals, it seemed expedient to observe, in the first place, the principal phenomena of apnœa in its least complicated form—namely, when produced by simply depriving the animal of air.

The principal facts to which attention was directed during the progress of the apnœa thus induced were—

The duration of the respiratory movements;

The duration of the heart's action.

The duration of the heart's action was observed—

(*a*) In relation to the duration of the respiratory movements.

(*b*) In relation to the time after the stoppage of the breathing.

From the experiments performed, it appeared that in the dog the average duration of the respiratory movements, after the animal has been deprived of air, is 4 min. 5 sec., the extremes being 3 min. 30 sec. and 4 min. 40 sec. The average duration of the heart's action is 7 min. 11 sec., the extremes being 6 min. 40 sec. and 7 min. 45 sec.

From these experiments, it appears that on an average the heart's action continues for 3 min. 15 sec. after the animal has ceased to make respiratory efforts, the extremes being 2 min. and 4 min. respectively.

Rabbits, on an average, ceased to make respiratory efforts in 3 min. 25 sec. Their heart's action stopped in 7 min. 10 sec.; consequently the interval between the last respiratory effort and the cessation of the heart's action was 3 min. 45 sec.

The next question investigated was—the period after the simple deprivation of air at which recovery is possible, under natural circumstances, without the aid of any artificial means of resusitation.

The experiments performed led to the conclusion that a dog may be deprived of air during 3 min. 50 sec., and afterwards recover without the aid of artificial means; that a dog is not likely to recover, if left to itself, after having been deprived of air during 4 min. 10 sec.

The force of the inspiratory efforts during apnœa was observed in the experiments to be so great that it was determined to measure them. They were found to be capable, in the dog, of raising a column of mercury four inches. It appeared, moreover, that their force increases up to a certain period.

In other experiments, plaster of Paris, and even mercury, were thus drawn upwards into the minute bronchial tubes.

It is easy to understand, therefore, how foreign bodies may be drawn into the lungs, in cases of drowning, and the importance of this fact in the consideration of the pathology and treatment of apnœa.

The committee next passed on to the subject of drowning.

The first question investigated was—For what period can an animal be submerged, and yet recover without the aid of artificial means?

It was found, as the result of numerous experiments on dogs, that in striking contrast to the previous ones, 1½ minute's immersion in water suffices to destroy life.

Other experiments satisfactorily showed that the difference of time between simple apnœa and that by drowning is not due to submersion, or to depression of temperature, or to struggling, but that it is connected with the fact, that in the one case a free passage of air out of the lungs, and of water into them, is permitted; in the other, the exit of air and the entrance of water are prevented.

There can be no doubt, from other considerations put forward, that although both these circumstances are concerned in producing the difference observed, yet that it is mainly due to the entrance of water and the effects thereby produced.

The treatment of apnœa was next considered.

For conclusions respecting artificial respiration, the committee refer to the second portion of the Report.

Many other methods of resuscitation which have been recommended were employed, including actual cautery, venesection, cold splash, alternate application of hot and cold water, galvanism, puncture of the diaphragm.

Although some of the above means were occasionally of manifest advantage, no one was of such unequivocal efficacy in a sufficient number of cases as to warrant the committee in specially recommending its adoption.

The experiments upon the dead subject were made with a view to determine the value of the various methods which have been employed for alternately compressing and expanding the cavity of the chest in such a manner as to imitate the natural movements of the thoracic walls in breathing. The following methods have been investigated:—

1. Pressure exerted by the hands on the anterior wall of the thorax, the body being in the prone posture. Such pressure has for its object, to expel a portion of the air contained in the chest; on relaxing the pressure, the chest expands and air enters.

2. The postural, or so-called "ready" method, described by Dr. Marshall Hall, consists essentially in "turning the body gently on the side and a little beyond, and then briskly on the face alternately;" and in making pressure along the back of the chest each time the body is brought into the prone position.

3. The method of Dr. Silvester, in which the action of the pectoral and other muscles passing from the shoulders to the parietes of the chest in deep inspiration is imitated. An inspiratory effort is produced by extending the arms upwards by the sides of the head; on restoring them to their original position by the side of the body, the expanded walls are allowed to resume their previous state, and expiration takes place, the quantity of air expelled being in proportion to that which had been previously inspired.

It being necessary to measure the flow of air in and out of the respiratory cavity under conditions of pressure closely resembling those which exist in natural respiration, no means of measurement could be used, which, in its working, would offer any appreciable resistance to the passage of air. With this consideration in view, an instrument designed by Dr. Sanderson was employed.

GENERAL RESULTS.

1. As regards the volume of air which can be expelled from the thorax by compression of its walls, and inspired by the elastic expansion consequent on relaxation of the pressure, it was found —

(*a*) That pressure by both hands on the lower third of the sternum in the adult male subject usually displaced from 8 to 10 inches of air.

The pressure actually exerted amounted to about 30 lbs. It was, therefore, not greater than might be safely applied to the living subject. The volume of air expelled varied from 8 cubic inches to 15 cubic inches.

(*b*) That pressure made in the same manner on the upper part of the sternum usually displaced 2 or 3 cubic inches less than pressure on the lower part.

(*c*) That pressure exerted by one hand on the upper part, by the other on the lower part of the sternum, produced about the same results as were observed in *a*.

In this case, the whole amount of pressure did not exceed that exerted in *a*.

(*d*) That the pressure of a weight laid on the lower third of the sternum produced similar results according to its amount.

(*e*) That lateral pressure exerted on the ribs or costal cartilages of both sides simultaneously was in no instance more effectual.

(*f*) That compression by a broad bandage encircling the chest, the ends of which were crossed over the sternum, and drawn in opposite directions by two persons, produced no greater effect than pressure with the hands on the sternum or sides.

2. As regards the whole amount of exchange of air produced by the method of Dr. Marshall Hall, "to imitate respiration," it varied much, according as the subject was favorable or the contrary; sometimes not exceeding a few cubic inches, but never exceeding 15 cubic inches.

3. As regards Dr. Silvester's method, it was found, that on extending the arms upwards, a volume of air was inspired into the chest, which varied, in different subjects, from 9 to 44 cubic inches, and it was observed that the results obtained in successful experiments on the same body were remarkably uniform, in which respect, as well as in their amount, they contrasted with those obtained by the method of Dr. M. Hall. On restoring the arms to the side, the quantity of air expelled was generally nearly equal to that previously inspired, occasionally less.

In the treatment of apnœa generally, the committee offer the following suggestions:—

That all obstruction to the passage of air to and from the lungs be at once, so far as is practicable, removed;—that the mouth and nostrils, for example, be cleansed from all foreign matters or adhering-mucus.

That in the absence of natural respiration, artificial respiration by Dr. Silvester's plan be forthwith employed in the following manner:—The body being laid on its back (either on a flat surface, or, better on a plane inclined a little from the feet upwards), a firm cushion or some similar support should be placed under the shoulders, the head being kept on a line with the trunk. The tongue should be drawn forward so as to project a little from the side of the mouth. Then the arms should be drawn upwards until they nearly meet above the head (the operator grasping them just above the elbows), and then at once lowered and replaced at the side. This should be immediately followed by moderate pressure with both hands upon the lower part of the sternum. This process is to be repeated 12 or 14 times in the minute.

That if no natural respiratory efforts supervene, a dash of hot water (120° Fahr.) or cold water be employed, for the purpose of exciting respiratory efforts.

That the temperature of the body be maintained by friction, warm blankets, the warm bath, etc.

In the case of drowning, in addition to the foregoing suggestions, the following plan my be in the first instance practised: —Place the body with the face downwards, and hanging a little over the edge of a table, shutter, or board, raised to an angle of about 30 degrees, so that the head may be lower than the feet. Open the mouth and draw the tongue forward. Keep the body in this posture for a few seconds, or a little longer if fluid escapes. The escape of fluid may be assisted by pressing once or twice upon the back.

[Signed by the Committee of eight, C. J. B. WILLIAMS, Ch'm.]

Dr. C. J. B. Williams said, that if the subject of suspended animation and its treatment appeared to be one of the greatest importance when the committee were appointed for its investigation, the result of their labors did not make it less so; for during their researches, several new points of both physiological and practical interest had arrested their attention. The Report just read contained a large mass of facts bearing on the subject, and these facts would be fully appreciated when they should be maturely considered; but the members of the committee thought it might be acceptable to the Society if one of their body were to give a short summary of some of the most striking results. He (Dr. Williams) had been requested to do this since he entered the room, and not having been previously aware of the office which would devolve on him, he was not prepared to go fully into details; but he believed that he was sufficiently acquainted with the general results of the experiments to be enabled to give a summary of their most important features. He would premise that he could take no merit to himself with regard to the experiments which had been so ingeniously devised and laboriously carried on by other members of the committee. He had been present at very few of the experiments themselves; but, as chairman of the committee, had merely assisted in receiving and completing the reports from the sub-committees. The committee, having to consider the subject of "Suspended Animation," directed their inquiries to that kind of interference with life which results from stoppage of the breath in suffocation, strangulation, and drowning.

The first series of experiments was to investigate the result of simple apnœa, or stoppage of the breath; and for this purpose the trachea of animals was opened, and a tube inserted so as to command the supply of air; and this tube being furnished with a stop-cock could be closed, and the results noted, especially these:—After the closure of the tube, 1, how long respiratory efforts continue; 2, how long the heart's action continues; 3, how long the heart beats after the breathing efforts cease. The experiments show a considerable variety of result; but, as a general average, it may be stated that in dogs efforts at breathing continued a few seconds more than four minutes after the closure of the tube; and the heart's action three minutes and a-quarter longer. The duration and force of these respiratory efforts, in an animal deprived of air, were not more remarkable than important, as indicating the period within which an animal deprived of air could recover; and this was found to be almost, but not quite, as long as the duration of these efforts—

that is to say, a dog deprived of air four minutes only, would recover; but if the exclusion of air lasted ten seconds longer, he did not recover. The extraordinary force of these struggles for breath was shown by plunging the end of the tube into mercury; when it was found that the inspiratory effort sometimes raised a column of four inches of mercury, and, if the tube was shorter, would draw the quicksilver in considerable quantites into the bronchial tubes and air-cells of the lungs.

The next subject of investigation was suspended animation from drowning; and here the experimenters soon found a remarkable difference in the greater rapidity of the death, and the shorter time during which life is recoverable. An animal simply deprived of air for four minutes may recover; but one immersed in water for one minute and a-half is irrecoverably dead. Recovery took place in several cases where the immersion lasted one minute and fifteen seconds; but fifteen seconds more made all the difference. The experimenters proceeded to search into the cause of this peculiarly destructive operation of drowning, as compared with simple privation of air; and very soon they were enabled to trace it to the action of the water itself, forcibly drawn into the lungs by the respiratory struggles of the animal. Two dogs were plunged into water, one having its trachea closed by a stop-cock at the moment of immersion. The dog with the trachea free was taken out in two minutes, irrecoverably dead. The other, with the trachea closed, was taken out at the end of four minutes; the trachea was opened, and in the course of a few seconds the animal began to gasp, and soon recovered. Another mode of diminishing the inspiratory struggles of the animal was by stupefying it with chloroform before immersion in water, and it was actually found that recovery took place after two minutes and fifteen seconds immersion. On this point he (Dr. Williams) adverted to a popular opinion, that it was more difficult to drown a drunken man than one who is sober, as having some foundation on this fact, that insensibility of any kind retards the fatal influence of drowning by diminishing those violent struggles for breath which, by forcing water into the lungs, soon put the case beyond recovery. But nothing so fully pointed out the extent and nature of the fatal influence of water in the lungs as the appearance of these organs in drowned animals as compared with those killed by simple apnœa. In the latter, the air-passages remained free from all secretion or effusion, and the lungs themselves were light and buoyant, and contained remarkably little blood. Now, this is contrary to what is generally described as the state of the lungs in asphyxia; and probably in ordinary cases, where death is not sudden, but prolonged, more or less engorgement may take place. But here there was no engorgement or obstruction, and it is not wonderful that animals would recover more readily. But with drowned animals not only were all the air-passages choked with frothy fluid, and that fluid generally more or less bloody, but the whole lungs were always highly engorged with blood, so that they were heavy, dark colored, pitted on pressure, and on being cut, exuded an abundance of blood-tinged fluid, with many air-bubbles in it.

On this subject he would make two remarks, on his own responsibility, apart from his office in the committee. One was, How opposed these observations and conclusions are to those many years ago propounded by Goodwyn, in his treatise on Suspended Animation, whose opinions have generally been adopted to the present time. Goodwyn concluded, from his observations, that water never to a hurtful extent enters the lungs of the drowned, and he depricated the popular practise of hanging up a drowned person by the heels to let the water run out. He (Dr. Williams) was by no means sure that, as Dr. Goodwyn was certainly wrong in his pathology, some modification of the popular practice may not be beneficial.

The other remark related to the mode in which the water which got into the lungs of the drowned proved so rapidly and extensively injurious. No doubt much was due to its mechanical pressure on the tubes and cells, forming an impervious barrier to the readmission of air; but this would not account for the extraordinary increase of blood in the lung, and its transudation into the air-tubes. He believed the injurious influence of water to be due to its chemical power of acting by endosmosis on the blood within the capillaries of the lungs, swelling up and bursting the blood-corpuscles, and causing their rapid accummulation in the organ, and their extravasation into the bronchial tubes. This was a subject for further experimental investigation, and he thought it one of great importance, as bearing on the action of water as a noxious or a therapeutic agent.

He would not detail the various means of resuscitation which were tried by the committee, but the results of the trials were not such as to induce the committee to recommend them strongly for general adoption. Various instructive experiments were made on different modes of performing artificial respiration, and the most conclusive of these had reference to the so-called "ready methods" of Dr. Marshall Hall and Dr. Silvester. One of their committee (Dr. Sanderson) contrived the apparatus on the table for measuring the air which could be forced out of it into the lungs of a dead body by these methods of artificial respiration; and the general result was, that by Dr. Hall's method the quantity of air moved in and out of the lungs rarely reached nine cubic inches, and never exceeded fifteen; whereas, by Dr. Silvester's plan, an interchange of forty cubic inches was effected; and when this method was further improved by alternating the drawing up of the arms, with depressing them, and with pressure on the lower part of the sternum, the expelled air was as much as fifty cubic inches. So far, then, as these experiments go, they show a great superiority of Dr. Silvester's over Dr. Marshall Hall's "ready method."—*Boston Med. & Sur. Jour.*

